# The High-Effective Catalytic Degradation of Benzo[a]pyrene by Mn-Corrolazine Regulated by Oriented External Electric Field: Insight From DFT Study

**DOI:** 10.3389/fchem.2022.884105

**Published:** 2022-06-02

**Authors:** Tairen Long, Haiyan Wan, Jianqiang Zhang, Jie Wu, Jin-Xia Liang, Chun Zhu

**Affiliations:** ^1^ School of Chemistry and Chemical Engineering, Guizhou University, Guiyang, China; ^2^ Guizhou Fukangren Pharmaceutical Co., Ltd, Guiyang, China

**Keywords:** benzo[a]pyrene, Mn-corrolazine, catalytic oxidation, density functional theory calculations, oriented external electric field

## Abstract

The degradation of BaP into hydroxybenzo[a]pyrene by Mn-corrolazine and its regulation by an oriented external electronic field (OEEF) were systematically studied using first-principle calculations. Extensive density function calculations showed that the degradation of BaP into hydroxybenzo[a]pyrene by Mn-corrolazine occurs *via* a three-step process in the absence of OEEF, in which a more toxic and stable epoxide intermediate is generated. However, upon application of OEEF along the intrinsic Mn-O reaction axis, the degradation of BaP into hydroxybenzo[a]pyrene is greatly simplified. The negative charge on the terminal O atom of Mn-OO corrolazine increases with an increase in the OEEF intensity. As the intensity of the OEEF increases over 0.004 a.u., the negatively charged terminal O atom has the ability to directly abstract the positively charged H atom of BaP and the degradation of BaP into hydroxybenzo[a]pyrene can be completed *via* a one-step process, avoiding the production of more toxic epoxide intermediates.

## 1 Introduction

With the development of modern industry, the use of fossil fuels and discharge of soot, flue gas and industrial wastewater have resulted in serious environmental pollution from polycyclic aromatic hydrocarbons (PAHs) (Paulik et al., 2016; Poater et al., 2018; Wu et al., 2018; Yan et al., 2021). As a representative of carcinogenic PAHs, benzo[a]pyrene (BaP) bearing five fused aromatic rings, which is classified as a Group I “human carcinogen” by the World Health Organization (WHO) International Agency for Research on Cancer IARC (2021), has caused irreversible damage to air, water and soil (Lemieux et al., 2015; Strobel et al., 2015; Roberts et al., 2016). Moreover, BaP is persistent organic pollutant, which poses a serious threat to global food security (Średnicka et al., 2021). Seafood with increasing BaP content increases the risk of cancer (Wang et al., 2020) and BaP in roast meat, smoked meat and fried foods will cause serious damage to animals and human organs, such as the liver and kidneys (Takeshita and kanaly, 2019; Iko Afé et al., 2020; Cunha et al., 2021; Goedtke et al., 2021; Mertens et al., 2021; Ge et al., 2022). Very recent studies (Kostoff et al., 2020) have indicated that people exposed to increased levels of BaP will lead to degradation or dysfunction of the immune system and people will become more susceptible to 2019-nCoV. Therefore, to eliminate the toxicity of BaP, it is essential to study its degradation.

BaP is mainly degraded *via* chemical oxidation, photo-oxidation, microbial degradation and bioaccumulation in the natural environment (Wilson and Jones, 1993; Rubio-Clemente et al., 2018). Among them, the use of bacteria or fungi to biodegrade BaP has received a lot of research attention (Kou et al., 2009; Kuppusamy et al., 2016; Ping et al., 2017; Qin et al., 2017; Delsarte et al., 2018; Gan et al., 2018; Harry-Asobara and Kamei, 2019; Ostrem Loss et al., 2019; Sampaio et al., 2019; Tian et al., 2019), but this approach has a series of drawbacks, such as low catalytic efficiency and difficulty in cultivating suitable bacteria (Wang et al., 2012; Wongwongsee et al., 2013). From the perspective of utilizing solar energy, photocatalysis is one of the most attractive BaP degradation methods reported to date (Kou et al., 2009). BaP can be directly photodegraded upon adsorbing solar energy (Miller and Olejnik, 2001) or degraded using sensitized photochemical reactions (Mill et al., 1981; Fasnacht and Blough, 2002). Recently, the group Luo et al. (2015); Luo et al. (2018) has reported for the first time that natural porphyrins can promote the photoconversion of BaP in water into quinones using singlet oxygen generated *via* a photocatalytic detoxification reaction (Zang et al., 2007). In addition, the possible oxidative degradation mechanisms and pathways of BaP in the atmosphere have been explored by Dang et al. (2015). However, despite the involvement of transition metals in many catalytic processes (Fujihara and Tsuji, 2019; Lin et al., 2021; Parmar et al., 2021; Sánchez-López et al., 2021; Yang et al., 2021; Zhou et al., 2021; Zhou et al., 2022), the use of transition metal complexes as non-sacrificial oxidants for the degradation of BaP has not been reported in the literature.

Our group has recently reported that Mn-corrolazine can activate oxygen in the air *via* an electron spin-flip mechanism under visible light irradiation, which forms a singlet [Mn]-O-O species with terminal radical characteristics (Zhu et al., 2018). In fact, corrole has been widely used in many fields (Zhu and Liang, 2015; Zhu et al., 2016; Li and Cao, 2018; Lvova et al., 2018; Liang et al., 2019; Zheng et al., 2020; Dedic et al., 2021; Li et al., 2021). Very recently, oriented external electric fields (OEEFs) have been extensively studied as a ‘smart agent’ by Shaik et al. (2016); (Ramanan et al., 2018; Wang et al., 2018); Shaik et al. (2020). They found that the interaction between OEEFs and dipole moments can change the electron transfer process and enhance the ionicity in the direction of the “reaction axis”, resulting in the regioselectivity of the reaction. Furthermore, our group has also found that OEEFs can effectively regulate the catalytic activity of metal-corrolazines (Wang et al., 2021; Zhu et al., 2021). Therefore, in order to discover a new effective and environmentally-friendly strategy for the degradation of BaP, we have explored the degradation mechanism of BaP to form hydroxybenzo[a]pyrene using Mn-corrolazine involving a [Mn]-O-O species. We further studied the regulation of OEEF on the degradation process. Based on considerable theoretical calculations, we have discovered the reasonable reaction process and OEEF regulation mechanism, which provide a theoretical foundation for further experimental research on the degradation of BaP using Mn-corrolazine regulated by OEEF.

## 2 Computational Methods

The B3LYP density functional method (Lee et al., 1988; Stephens et al., 1994) in combination with an effective core pseudopotential basis set (LANL2DZ) for the Mn atom and 6-31G(d) basis set for all other atoms (B1) was used for all geometry optimizations and frequency calculations performed in the Gaussian 16 package (Frisch et al., 2016). The energies were refined using single-point calculations (Stuyver et al., 2020) utilizing the B3LYP functional with D3BJ dispersion correction (Grimme et al., 2011) coupled with 6-311+G (d,p) (Andersson and Uvdal, 2005) and aug-cc-pVQZ (Mn) (Kendall and Harrison, 1992), i.e., B3LYP-D3(BJ)/6-311+G (d,p), aug-cc-pVQZ (Mn) level of theory (B2). Zero-point energy (ZPE) corrections were taken from the B3LYP/6-31G(d), LANL2DZ (Mn) level of theory. Twelve structures formed by [Mn]-O-O adsorbing different H atoms from BaP were fully optimized to obtain the most stable structure. Based on this structure, the degradation of BaP was studied in which the reactant complexes (RC), transition states (TS), intermediates (IM), and products (P) were fully optimized without any symmetry constraints. Frequency calculations were carried out at the same level of theory in order to assess the nature of a stable point on the potential energy surface and estimate the thermodynamic properties. Moreover, intrinsic reaction coordinate (IRC) analysis was used to further confirm the TS correlating to their corresponding RC and P.

**SCHEME 1:**
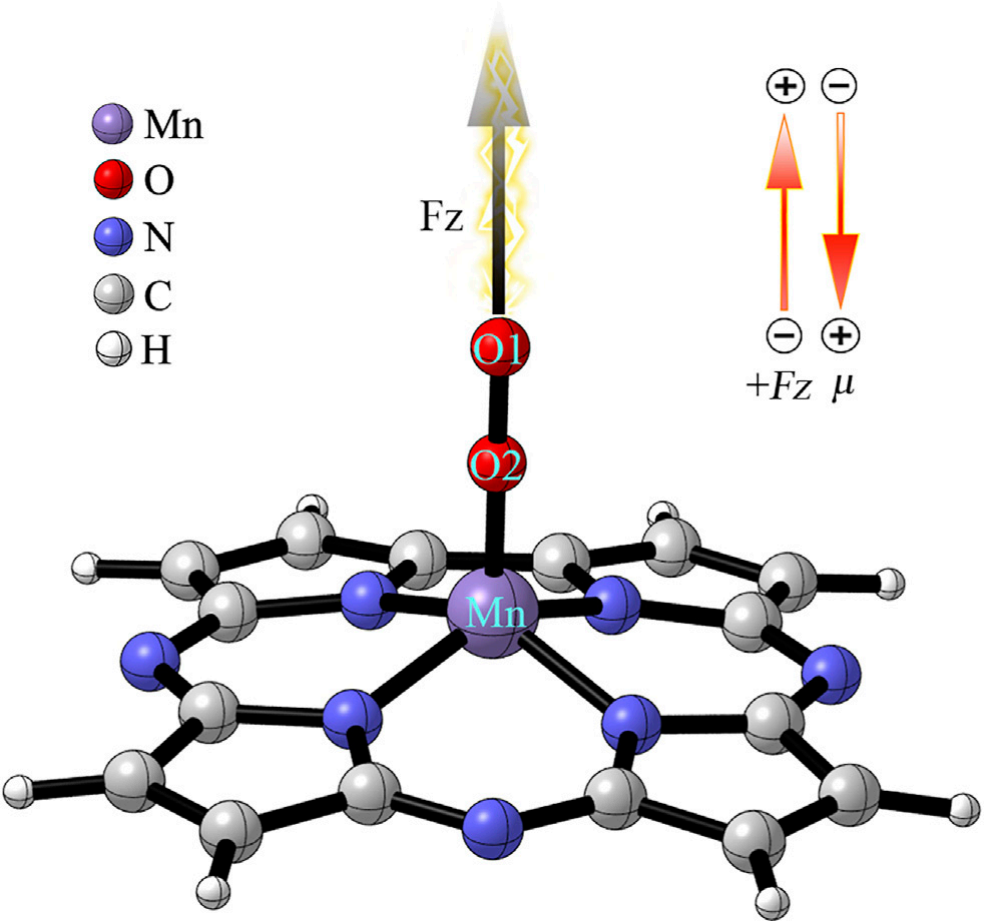
Definitions of the OEEF. F_Z_ is along the intrinsic Mn-O reaction axis perpendicular to the corrolazine ring. The inset indicates the definition of a positive OEEF (Fz > 0) and the stabilizing orientation of the dipole moment (*μ*_*z*_).

To investigate the effect of OEEF on the reaction, the OEEF Fz along the intrinsic Mn-O reaction axis perpendicular to the corrolazine ring was applied using the keyword “field = M ± N”, as shown in Scheme 1. The positive direction of the electric field vector in Scheme 1 follows the Gaussian 16 convention. In addition, the electric field strength of Fz was in the range of 0.001–0.01 a.u. (1 a.u. = 51.4 V/Å).

## 3 Results and Discussion

### 3.1 Reactive Site Screening

**FIGURE 1:**
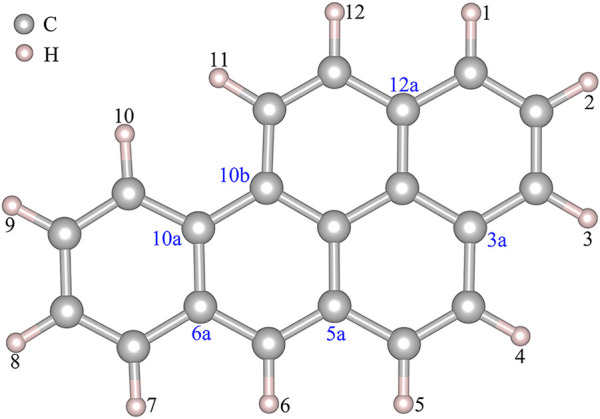
Partial atomic numbers of BaP.

**FIGURE 2:**
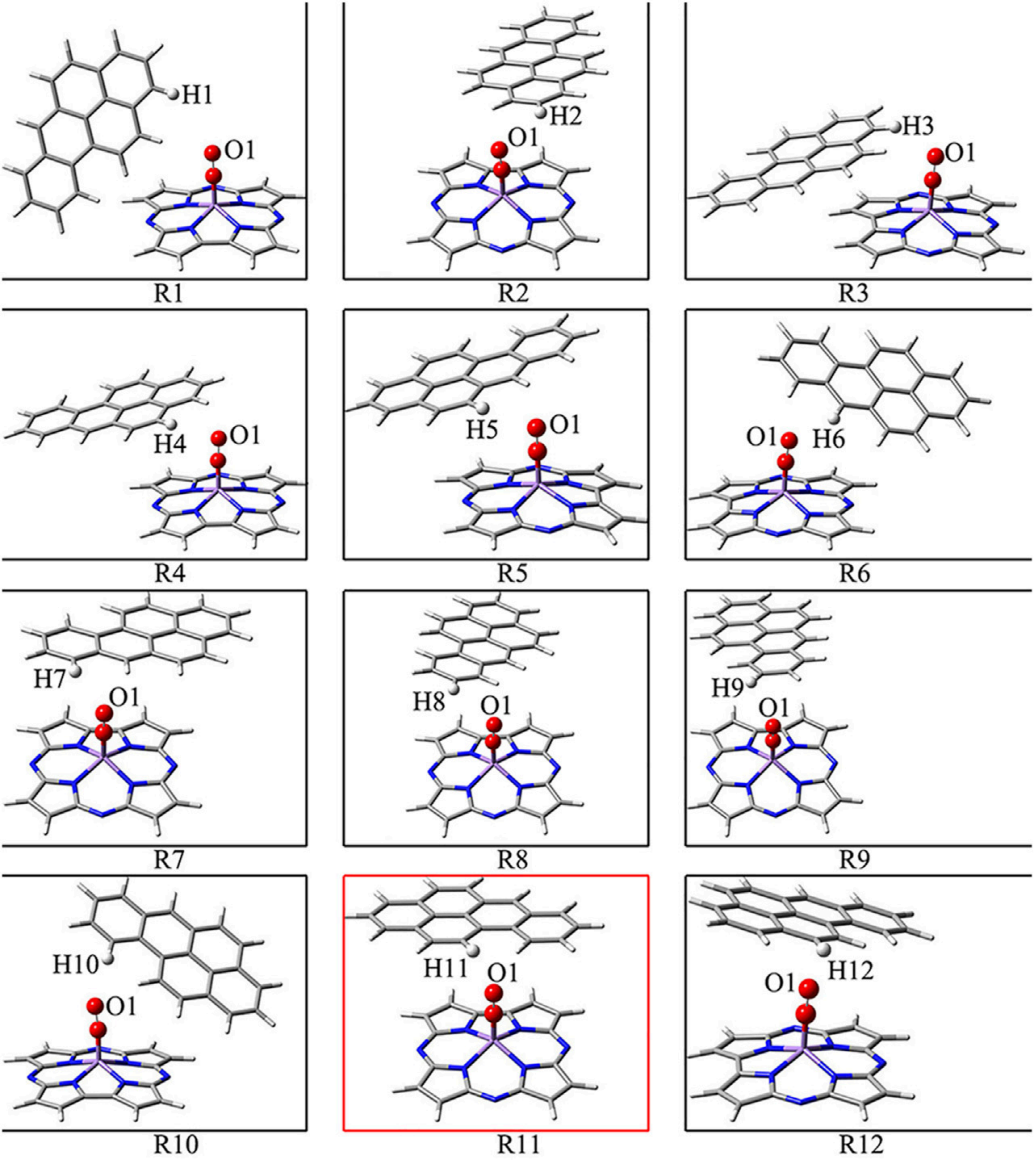
Optimized structures of the different reactant complexes.

Figure 1 shows that BaP has 12 H atoms in different chemical environments and in order to find the most reasonable reaction site, we optimized the different RC formed by these 12 different H atoms in BaP and the [Mn]-O-O corrolazines, respectively. Figure 2 and Table 1 show that BaP and [Mn]-O-O corrolazine form weak interaction complexes, in which the bond lengths of d_O1-HX_ (X = 1, 2, 3, ..., 12), where the numbers refer to the serial numbers of the H atom, are very similar in the range from 2.537 to 2.845 Å, and the bond length of d_O1-H11_ was the smallest, indicating its interaction was the strongest. Similarly, the energies of these structures are very similar, in which the difference between the highest energy and the lowest energy was only 1.07 kcal/mol, which originates from the weak interaction formed between BaP and [Mn]-O-O corrolazine. In addition, the energy of structure **11** was the lowest, again proving that it was the most stable. Furthermore, Table 1 shows the bond lengths of Mn-O and O-O were 1.617 and1.265 Å, respectively, which are similar to the results obtained in our previous study (Zhu et al., 2018), indicating that the [Mn]-O-O moiety in structure **11** has strong oxidation ability. Therefore, we chose structure **11** as the RC to further explore the degradation of BaP.

**TABLE 1:** **|**O1-HX (X = 1, 2, 3, ..., 12), Mn-O, O-O bond lengths (Å), relative electronic energies and zero-point energies (ΔE, ΔE_0_, kcal/mol).

Structure	d_O1-HX(X=1, 2, 3, ......,12)_	d_Mn-O_	d_O-O_	ΔE	ΔE_0_
R1	2.697	1.617	1.264	0.218	0.166
R2	2.776	1.617	1.262	0.792	0.683
R3	2.671	1.617	1.264	0.319	0.263
R4	2.599	1.617	1.263	1.015	0.907
R5	2.608	1.618	1.264	0.953	0.838
R6	2.703	1.619	1.263	0.945	0.809
R7	2.648	1.617	1.264	0.342	0.288
R8	2.845	1.617	1.262	0.725	0.604
R9	2.778	1.617	1.261	0.802	0.684
R10	2.676	1.617	1.263	0.705	0.647
R11	2.537	1.617	1.265	0	0
R12	2.563	1.618	1.264	1.068	0.958

### 3.2 Reaction Mechanism of [Mn]-O-O Catalysed Oxidation of BaP Into 11-OH-BaP

**FIGURE 3:**
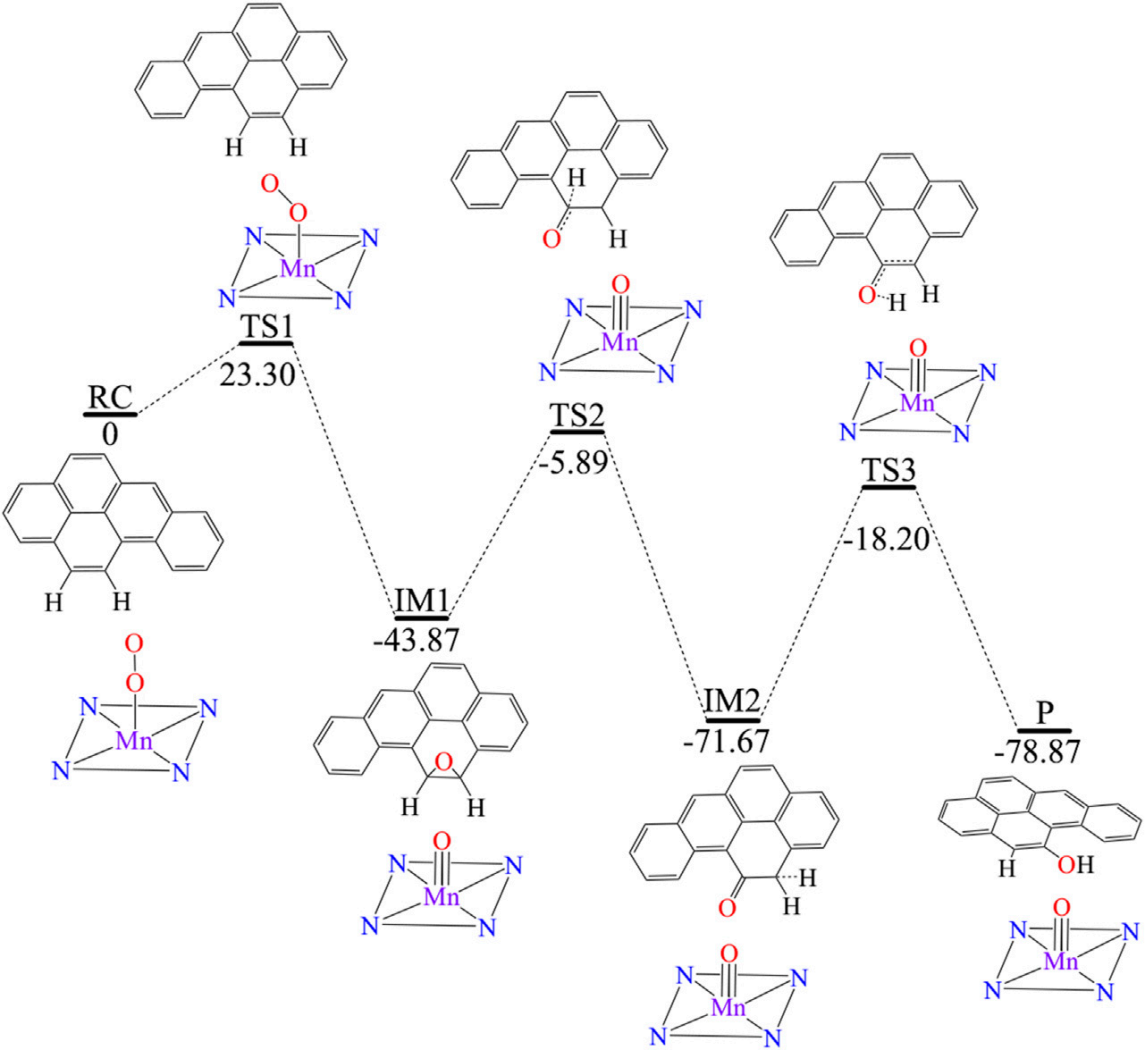
Predicted reaction pathway for the generation of 11-OH-BaP from BaP *via* [Mn]-O-O corrolazine catalytic oxidation in the absence of an electric field.

Figure 3 shows [Mn]-O-O corrolazine catalyses the degradation BaP into 11-hydroxybenzo[a]pyrene (11-OH-BaP) *via* three steps: 1) epoxidation, 2) hydrogen transfer and 3) rearrangement. The first epoxidation step in which 11,12-epoxybenzopyrene (IM1) was generated from BaP. The O-O bond in the [Mn]-O-O moiety was tilted and its terminal O1 attacks the C12 atom in the BaP moiety. The BaP moiety then rotates and its C11 atom attacks the terminal O1 atom to form 11,12-epoxybenzopyrene with a reaction activation energy of 23.3 kcal/mol. During this step, the hybridization of C11 and C12 changes from sp^2^ to sp^3^. Following the epoxidation step, IM2 was formed *via* a hydrogen transfer step. In this step, the C11-O1 bond was broken and at the same time, the C11-H11 was broken with H11 being transferred to C12 and C11 returns to sp^2^ hybridization. The activation energy of this step was up to 37.98 kcal/mol. The third step was an H atom rearrangement to form 11-OH-BaP (P), in which one H atom on C12 is transferred to O1, followed by a rebound reaction to form P with C12 returning to sp^2^ hybridization. In addition, the activation energy in this step was even higher (up to 53.47 kcal/mol). Using whole process analysis, P was the most stable with the lowest energy compared to RC, IM1 and IM2. However, as the activation energies in step 2 and step 3 are very high (37.98 and 53.47 kcal/mol respectively), it is difficult to reach the final product (P) *via* TS2 and TS3. Thus, the reaction will stop at the intermediate product, 11,12-epoxybenzopyrene, which is a carcinogen and even more toxic than BaP. Therefore, it is necessary to seek an efficient way to degrade BaP into the final non-toxic product, 11-OH-BaP.

### 3.3 One-step Degradation Mechanism Regulated by OEEF

#### 3.3.1 The Effect of OEEF on the RC and TS

To simplify the reaction process so that BaP can be degraded into 11-OH-BaP in one-step, thereby avoiding the production of highly toxic epoxybenzopyrene, we applied an OEEF Fz along the z-axis, which is the intrinsic Mn-O bond reaction axis perpendicular to the corrolazine ring, in order to regulate the degradation reaction process, as shown in Scheme 1.

**FIGURE 4:**
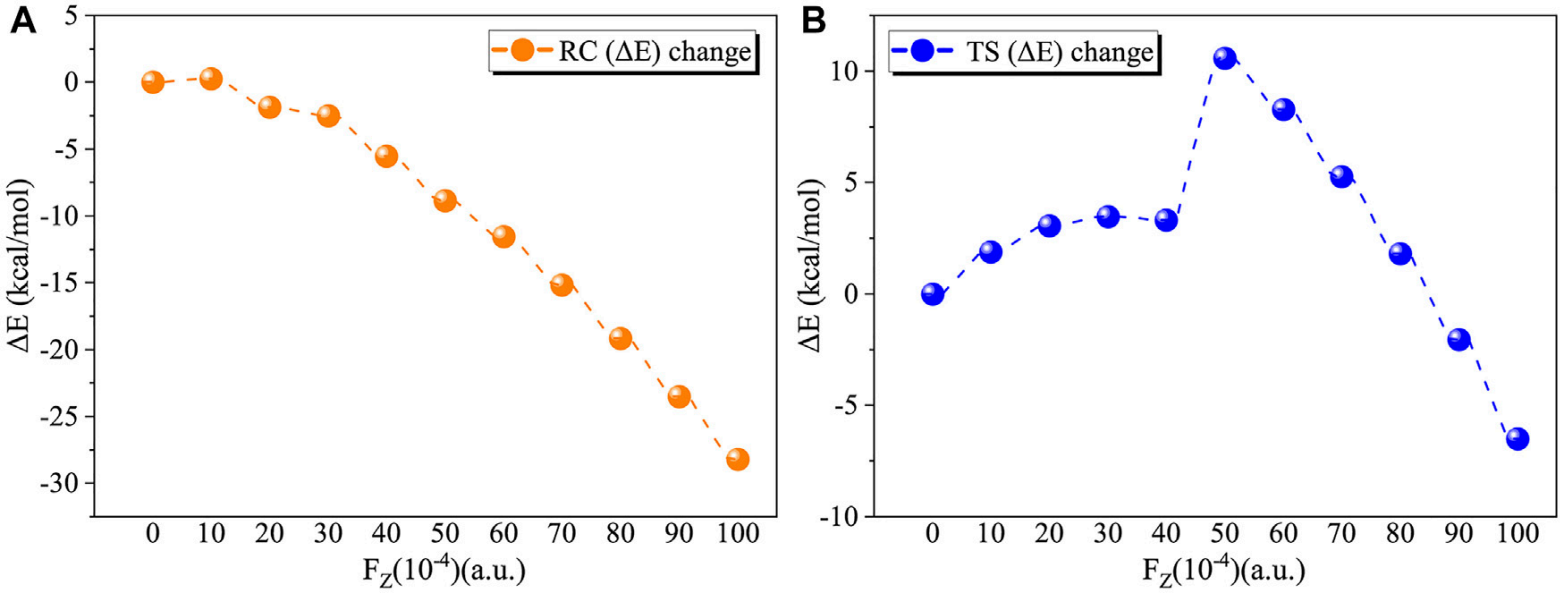
**(A)** Variation in the relative electron energy of the reactants (RC) with the electric field strength. **(B)** Variation in the relative electron energy of the transition states (TS) with the electric field strength.

We first considered the effect of the Fz on the stability of RC in the range of 0–0.01 a.u. Table 2 and Figure 4A show the application of the Fz increases the stability of the RC, in which the relative electronic energy increases upon increasing the electric field intensity. To further explore the reason why the stability of the RC becomes stronger due to the application of the Fz, we analysed the change in the dipole moment in the z-orientation of the RC upon changing the Fz. Table 2 shows the dipole moment in the z-orientation of the RC increases from |–2.19| D without OEEF to |–20.05| D in Fz = 0.01 a.u. Thus, the enhanced stability of the RC originates from the interaction between the increased dipole moment in the z-orientation and the Fz.

**TABLE 2:** **|** Variation in the relative electronic energy and dipole moment on the z-axis for the reactants (RC) and transition states (TS) under different electric field strengths in the range of 0–0.01 a.u. Units: ΔE, kcal/mol and *μ*_*z*_, D.

F_Z_ (10^−4^) (a.u.)	Complex (ΔE, *μ*_*z*_)			
	RC (ΔE)	RC (*μ*_*z*_)	TS (ΔE)	TS (*μ*_*z*_)
0	0	−2.19	0	5.07
10	0.26	−4.94	1.89	2.27
20	−1.86	−6.46	3.06	2.24
30	−2.51	−7.92	3.47	−1.35
40	−5.53	−10.98	3.32	−6.38
50	−8.84	−12.56	10.58	−9.68
60	−11.53	−13.51	8.28	−11.16
70	−15.14	−15.15	5.27	−13.02
80	−19.17	−16.81	1.81	−14.89
90	−23.52	−18.41	−2.05	−16.67
100	−28.19	−20.05	−6.50	−18.61

For the TS, the effect of the OEEF becomes complex, in which different Fz strengths exhibit different behaviour. Table 2 shows for Fz < 0.008 a.u., the OEEF destabilizes the TS upon increasing its relative electronic energy. However, further increasing the intensity of Fz to more than 0.008 a.u., the OEEF stabilizes the TS upon decreasing its relative electronic energy, as shown in the insert of Figure 4B. The change in the stabilization can be attributed to the repulsion between the OEEF and increasing dipole moment in the z-orientation of the TS, from 5.07 D without an electric field to 2.24 D in Fz = 0.002 a.u. However, further increasing with dipole moment results in the dipole moment in z-orientation flipping its direction at the critical Fz value of 0.003 a.u., and the repulsion between the OEEF and molecular dipole moment in the z-orientation becomes attractive. Thus, the OEEF stabilizes the TS.

#### 3.3.2 The Effect of the OEEF on the Reaction Mechanism

**FIGURE 5:**
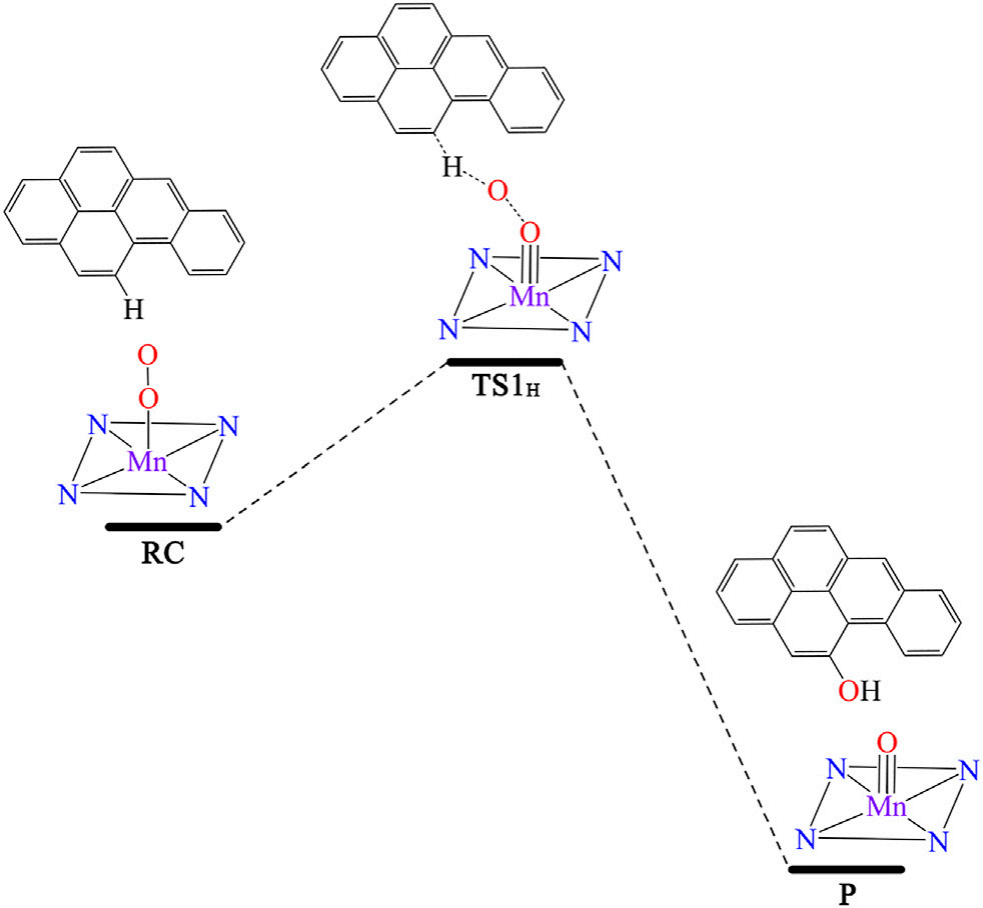
The one-step reaction process converting BaP into 11-OH-BaP.

We further investigated the effect of the OEEF on the mechanism of the degradation of BaP into 11-OH-BaP and discovered a very significant phenomenon. The reaction mechanism changes upon increasing the OEEF intensity, i.e., the degradation of BaP into 11-OH-BaP was simplified from a three-step process to a one-step process in which the H11 atom in BaP was directly transferred to the terminal O1 atom in [Mn]-O-O, followed by a rebound reaction of the resulting OH group back to BaP to form 11-OH-BaP, as shown in Figure 5. To further explore the reason for the change in the BaP degradation mechanism, we calculated the Mulliken charges of the atoms in the TS involved the H11 atom transfer step. Table 3 shows the Mulliken charge at O1 was –0.203 |e| without the OEEF. As the Fz strength increases from 0 to 0.004 a.u., the Mulliken charge of the O1 atom increases from –0.21 |e| to –0.30 |e|, increasing its attraction to the positively charged H11 atom. However, the BaP moiety in the reactant complex rotates continuously in this process, from almost parallel to the Mn-O bond to almost perpendicular to the Mn-O bond, so that the effect of the OEEF parallel to the Mn-O bond on BaP initially increases and then decreases. Therefore, the Mulliken charge on the H11 atom first increases from 0.175 |e| at Fz = 0 to 0.179 |e| at Fz = 0.002 a.u., and then decreases to 0.144 |e| at Fz = 0.004 a.u., which is the minimum value. In addition, the attraction of the negatively charged terminal O1 atom to the positively charged H11 atom was also weakened. Therefore, the reaction mechanism remains a three-step process, in which the terminal O1 atom is attracts to the C12 atom to form an epoxybenzopyrene intermediate. However, further increasing the OEEF intensity increases the negative charge on the terminal O1 atom to be more than 0.3 |e|. At the same time, the BaP moiety further rotates and deviates from direction parallel to the Mn-O bond, so the effect of the OEEF on BaP moiety was significantly strengthened and the positive charge on the H11 atom increases sharply. Moreover, as the positive charge of H11 exceeds that of C12, the interaction between H11 and the terminal O1 atom is stronger than that between C12 and O1, resulting in the TS to change from O1 attacking C12 to O1 attacking H11. Due to the change in the TS, the H11 atom can be directly transferred to the O1 atom to form 11-OH-BaP *via* a subsequent rebound reaction. Thus, the degradation of BaP into 11-OH-BaP was greatly simplified from a one-step to three-step process. Moreover, the simplified degradation reaction of BaP avoids the production of toxic epoxybenzopyrene.

**TABLE 3:** **|** Mulliken charges (|e|) of some atoms in the reactive centre of the transition state and activation energies ΔE_0_ (electronic energies + ZPE: kcal/mol) under an electric field.

F_Z_ (10^−4^) (a.u.)	O1	H11	C12	Activation energy
0	−0.203	0.175	−0.170	23.30
10	−0.216	0.171	−0.160	17.32
20	−0.210	0.179	−0.168	21.37
30	−0.230	0.166	−0.151	21.42
40	−0.300	0.144	−0.164	17.03
50	−0.357	0.276	−0.179	28.80
60	−0.362	0.280	−0.181	29.07
70	−0.368	0.285	−0.183	29.58
80	−0.374	0.291	−0.184	30.07
90	−0.379	0.294	−0.185	30.50
100	−0.386	0.299	−0.185	30.92

Analysis of the reaction activation energy of the degradation reaction of BaP into 11-OH-BaP upon the application of the OEEF shows its effect on the RC and TS was similar in the first step with the reaction activation energy varying slightly from Fz = 0 to Fz = 0.004 a.u. When the Fz was increased from 0.004 to 0.005 a.u., the reaction activation energy increases sharply from 17.03 to 28.80 kcal/mol due to the fundamental change in the TS. However, further increasing the OEEF from Fz = 0.005 to Fz = 0.01 a.u., the reaction activation increases slightly because the structure of the TS remains unchanged. Analysis of the whole reaction process shows that the reaction activation energy of the one-step process in Fz > 0.005 a.u. was higher than that in the first step of the three-step process and the activation energy of the one-step process was significantly less than those of the second and third step in the three-step process. Therefore, the regulation of the OEEF significantly simplifies the degradation reaction of BaP to an effective and environmentally-friendly process, which is of great significance to the food industry.

## 4 Conclusion

BaP is widely found in the natural environment and food, etc., and its degradation is of great significance for biosafety because of its carcinogenicity as a Group I “human carcinogen”. We have used Mn-OO corrolazine to degrade BaP into 11-OH-BaP in order to detoxify it. The degradation process occurs *via* a three-step process under field-free condition, i.e., epoxidation, hydrogen transfer and rearrangement, in which a more toxic epoxide intermediate is produced. Due to the high stability of the epoxide intermediate with an activation energy of up to 53.47 kcal/mol for further reaction, BaP is difficult to degrade into 11-OH-BaP using [Mn]-O-O corrolazine. However, the application of an OEEF along the intrinsic Mn-O reaction axis, which is more easily aligned in practical applications, greatly simplified the degradation of BaP into 11-OH-BaP. As the OEEF can effectively regulate the charge on the terminal O atom in [Mn]-O-O corrolazine, the H atom in BaP can be directly attracted by the terminal O atom to generate 11-OH-BaP *via* a subsequent rebound reaction. Thus, the complex three-step degradation process of BaP into 11-OH-BaP has been simplified into a one-step process, avoiding the generation of the more toxic and stable epoxide intermediate. This effective and environmentally-friendly degradation process will have a far-reaching impact on areas such as the environment and food industry.

## Data Availability

The original contributions presented in the study are included in the article/supplementary material, further inquiries can be directed to the corresponding authors.
